# Delayed Granulomas as a Complication Secondary to Lip Augmentation with Dermal Fillers: A Systematic Review

**DOI:** 10.1055/s-0042-1743524

**Published:** 2022-03-03

**Authors:** Lily Nguyen Trinh, Kelly Christine McGuigan, Amar Gupta

**Affiliations:** 1 Department of Otolarynology- Head and Neck Surgery, School of Medicine, Tulane University School of Medicine, New Orleans, Louisiana; 2 Department of Otolaryngology-Head and Neck Surgery, Massachusetts Eye and Ear Institution, Boston, Massachusetts; 3 Department of Otolarynology- Head and Neck Surgery, School of Medicine, Kimmel Medical College, Thomas Jefferson University Sidney, Philadelphia, Pennsylvania; 4 Department of Otolaryngology - Head and Neck Surgery, Facial Plastic Surgery, Los Angeles, California

**Keywords:** lip augmentation, fillers, nodules, granulomas, systematic review

## Abstract

**Introduction** Lip augmentation with dermal filler is rising in popularity. There are generally minimal side effects that are mild and transient. However, long-term complications may occur and include lumps, bumps, nodules, or granulomas. To better understand this uncommon but challenging outcome, we aim to perform a thorough systematic review of the published literature related to nodule or granuloma formation after cosmetic soft tissue augmentation of the lips.

**Methods** A search of published literature was conducted in accordance with PRISMA (Preferred Reporting Items for Systematic Reviews and Meta-Analyses) guidelines in April 2021 and included PubMed, ScienceDirect, Embase, Google Scholar, and Cochrane databases. The Medical Subject Headings (MeSH) terms used included the following terms: “lip filler,” “hyaluronic acid,” “lip injection,” “lip augmentation,” “silicone,” “poly-L-lactic acid,” “calcium hydroxyapatite,” “polymethylmethacrylate,” “complications,” “reaction,” “granuloma,” and “nodule.” All studies were reviewed by two independent reviewers. Any discrepancies were resolved by a third reviewer.

**Results** The initial search for filler-related nodules or granulomas yielded 2,954 articles and 28 were included in the final analysis containing 66 individual cases of lip nodules. All but one patient was female. The mean age was 50 years. Nodules presented on average 35.2 months or 2.9 years after initial treatment. Thirty-seven nodules underwent histological analysis, the majority of which identified the presence of a foreign-body granuloma. Silicone was the most reported filler used followed by hyaluronic acid. Most cases resolved following multiple treatments including oral antibiotics or steroids followed by surgical excision.

**Conclusion** Understanding the sequelae of lip augmentation with filler products allows clinicians to provide safe and effective treatment. Nodules that present months to years following dermal treatment may represent a foreign-body granuloma. A combination of oral antibiotics, intralesional or oral steroids, and surgical excision successfully treated the majority of cases in our study.

The lips are a central and defining aspect of an individual's face and have long been a target for enhancement and antiaging techniques. Due to the multitude of treatment modalities available today, treatments to the lips are becoming increasingly more common. There are numerous materials for lip augmentation including hyaluronic acid (HA), fat grafts, silicone, polyacrylamide, polymethylmethacrylate (PMMA), and poly-L-lactic acid (PLLA). Injections are used to treat lip asymmetry, lack of vermillion volume, vertical lip lines, downturned oral commissures, and/or an elongated upper lip, features which are normal consequences of aging. 1 Lip enhancement or augmentation with injectable fillers is rising in popularity as these treatments achieve rapid results with generally predictable outcomes. 2 This aesthetic treatment is also favorable due to its less invasive nature and minimal down time compared with surgical cosmetic procedures.

Soft tissue fillers are generally well-tolerated and major adverse events are rare. Mild side effects typically include limited and transient swelling, bruising, pain, and erythema. More severe complications include infection, nodule formation, vascular occlusion, and pigment changes. 3 Nodule formation may be characterized as early or delayed (4 weeks and later postinjection). 4 The incidence of delayed-onset nodules is uncommon and is reported to be 0.1 to 1.0%. 5 Delayed-onset nodules may be identified through histological analysis as foreign-body granulomas. If nodules or granuloma formation occur, they can be treated with intralesional steroids or hyaluronidase injections (for HA filler). However, if these treatments repeatedly fail, surgical excision may be required. 3 6

Delayed-onset granuloma formation has been a rarely cited complication of lip augmentation with filler. In this review, we aim to perform a thorough systematic review of the published literature related to nodule or granuloma formation after lip filler injections. Our goal is to determine the details associated with this complication including symptom description, time of onset, nodule characteristics, treatment, and outcomes. Since various terms for nodular formations have been used across providers, including lumps, bumps, nodules, or granulomas, these terms will be interchangeably used throughout this study unless otherwise specified. By gaining a better understanding of this rare but challenging outcome of lip fillers, providers can prevent future complications and provide patients reliable information regarding potential side effects.

## Methods

### Search Strategy

A systematic review of the published literature was conducted in accordance with the Preferred Reporting Items for Systematic Reviews and Meta-Analyses (PRISMA) guidelines to assess the association of granulomas with HA lip fillers. The literature search was performed in April of 2021, which included PubMed, ScienceDirect, Embase, Google Scholar, and Cochrane databases. The Medical Subject Headings (MeSH) terms used included the following terms: “lip filler,” “hyaluronic acid,” “lip injection,” “lip augmentation,” “silicone,” “poly-L-lactic acid,” “calcium hydroxyapatite,” “polymethylmethacrylate,” “complications,” “reaction,” “granuloma,” and “nodule.” The goal of the search was to compile and assess all of the published literature consisting of original articles including case reports, clinical trials, case series, and prospective case studies related to granuloma formation after filler lip augmentation.

### Study Selection

Studies were included if they met the following criteria: (1) described granuloma formation after lip filler, (2) were published between 2000 and 2021, and (3) included specific patient case information. Exclusion criteria included studies that: (1) were not published in English, (2) included nonhuman subjects, (3) were abstracts, communications, letter to the editor, or review articles, (4) did not report on location, onset, and treatment of granuloma, and (6) those discussing granulomas in other areas of the face.

### Data Abstraction

Titles and abstracts were screened for relevance by two separate reviewers (L.N.T. and K.C.M.). Of the selected articles that met the predetermined criteria, the full-text articles were retrieved and then independently reviewed by the two reviewers. Any discrepancies were resolved with the third investigator (A.G.). All studies which met the predetermined criteria were included in the final analysis. Relevant information from each included article was extracted which included author name, year of case reporting, patient age and sex, patient comorbidities, filler brand used, volume injected, site of injection, time of symptom onset after injection, presence of swelling or pain, number of nodules, nodule description, nodule duration/size, excision technique, treatment, and outcome. A summary of extracted information is depicted in Table 1 .

**Table 1:** Summary of the post-HA filler granuloma presentation and outcome

Author (first author)	Year	Country	Age	Sex	Brand	Site of injection (lips)	Onset (time after injection, mo)	Site of nodule (lips)	HAdase given?	Treatment start after Injection (mo)	Surgical treatment	Other treatments	Outcome
Kaczorowski 26	2019	Poland	52	F	NA	Both lips, NLF	24	Right buccal area	No	24	Excision	NA	Resolved
Alcântara 27	2017	Brazil	54	F	Perlane	Both	12	Both	No	NA	Excision	NA	Resolved
Rongioletti 15	2015	Italy	72	F	NA	Upper	36	Upper	No	36	Excision	NA	Resolved
Curi 10	2015	Brazil	65	F	Restylane	Upper	144	Upper	Yes	12	Punch biopsy	Oral steroids for 2 months	Remission after 3 months
Fernández-Aceñero 28	2003	Spain	48	F	Restylane	Upper	2	Upper	No	2	Punch biopsy	NA	Lost to follow-up
Edwards 29	2006	United States	74	F	Restylane	Both	6	Lower	No	6	Excision	NA	Resolved
Farahani 30	2012	United States	55	F	Restylane	Both	4	Upper	NA	4	Excision	NA	Resolved
			57	F	Restylane	Both	24	Lower	NA	24	Excision	NA	Resolved
			56	F	Restylane	Both	NA	Lower	NA	NA	Excision	NA	Resolved
Alijotas-Reig 8	2013	Spain	47	F	Restylane	Both lips, NLF, cheeks	15	Upper	No	15	Excision	Antibiotics (quinolones) and NSAIDs: no effect, oral prednisone + hydroxychloroquine (400 mg/d): nodules resolved	Resolved
			50	F	Restylane	Both	6	Upper	No	6	NA	NA	NA
Park 31	2011	South Korea	23	F	Unspecified	Lower	36	Lower	Yes	36	Excision	NA	Resolved
	2018	United Kingdom	24	F	NA	Upper	NA	Upper	No	NA	NA	NA	NA
			43	F	NA	Upper	NA	Upper	No	NA	NA	NA	NA
Alghonaim 32	2016	Canada	52	F	Restylane	Both	1	Lower	NA	36	Excision	NA	Resolved

Abbreviations: F, female; NA, not available or reported.

## Results

### Study Selection

**Fig. 1:**
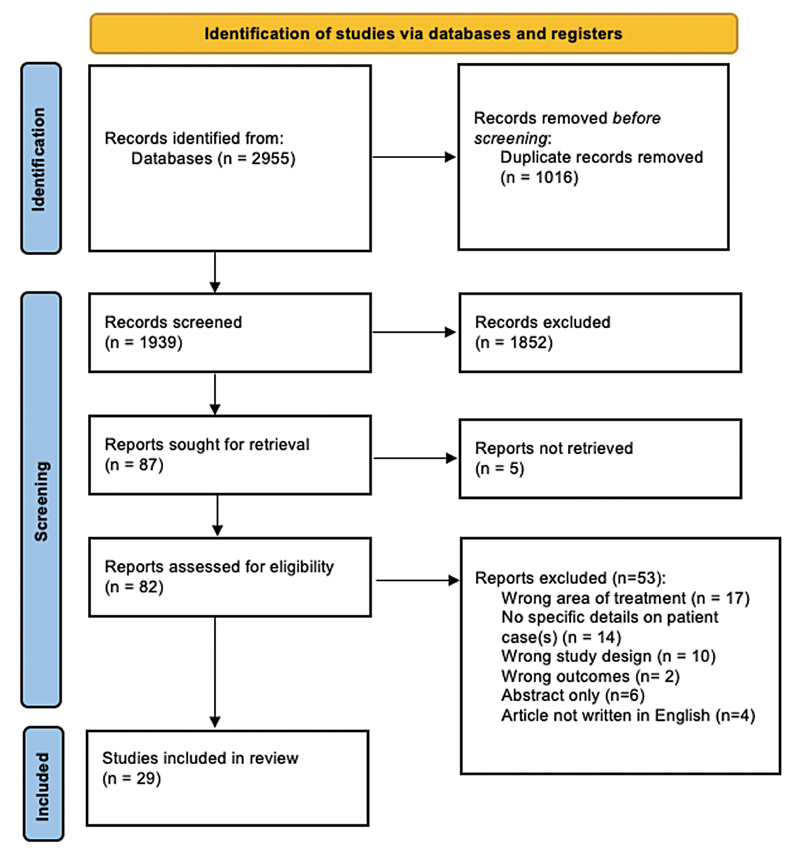
PRISMA 2020 flow diagram of screening process.

The initial search for filler-related nodules or granulomas yielded 2,955 articles. After removing 1,016 duplicates, 1,939 studies were screened by title and abstract. A total of 1,852 studies were eliminated based on the predetermined inclusion and exclusion criteria. Five articles were unable to be retrieved. The remaining 82 articles underwent full-text review. Fifty-three studies were excluded due to wrong treatment area of the face ( *n* = 17), do not include specific case information ( *n* = 14), wrong study design ( *n* = 10), wrong outcomes ( *n* = 2), abstract only ( *n* = 6), and published in a language other than English ( *n* = 4). A total of 29 articles were included in the final analysis. Fig. 1 demonstrates the breakdown of the literature search.

### Study Characteristics

**Fig. 2:**
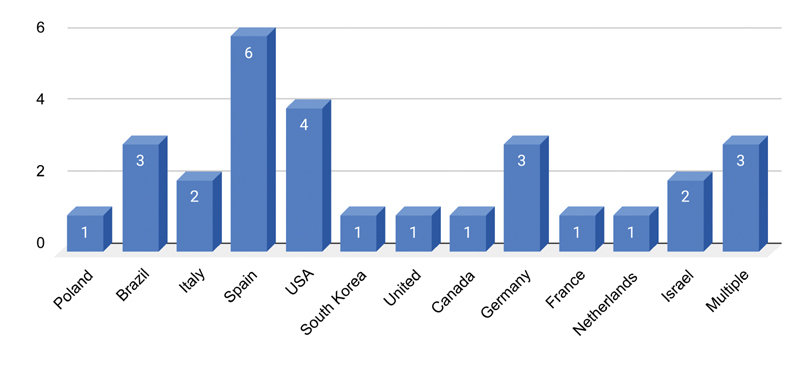
Country of study publication.

**Fig. 3:**
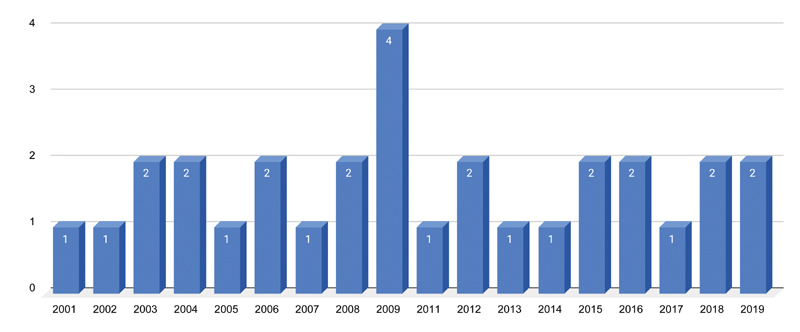
Number of study publication by year.

An overview of the study characteristics is summarized in Table 1 for HA and in Table 2 for non-HA. Of the 29 included studies, there were 68 cases reported on the incidence of nodule or granuloma formation following lip augmentation with dermal filler. This selection of 29 articles included 15 (51.7%) case reports (articles containing the description of one case) and 14 (48.3%) case series (articles evaluating more than one case). Most studies originated from Spain ( *n* = 6; 20.7%). Four were from the United States, and many other countries were also represented ( Fig. 2 ). The article publication years ranged from 2003 to 2019 ( Fig. 3 ).

**Table 2:** Summary of the post-non-HA filler granuloma presentation and outcome

Author (first author)	Year	Country	Age	Sex	Type of filler	Site of injection (lips)	Onset (time after injection, months)	Final treatment	Outcome
Wang 33	2018	United States	63	F	Silicone	Upper lip, NLF	36	Surgical	Resolved
Sanchis-Bielsa 12	2006	Spain	70	F	Collagen	Upper lip, NLF	12	Systemic steroids	Resolved
			43	F	Silicone	Both lips	30	Systemic steroids	Resolved
Schmidt-Westhausen 34	2004	Germany	56	F	Silicone	Lower lip	12	Surgical	Resolved
Hamilton 35	2008	France	58	F	PLLA	Both lips	18	Intralesional steroids	Resolved
Dijkema 36	2005	Netherlands	64	F	PLLA	Upper lip	14	Surgical	Not reported
Bigatà 37	2001	Spain	30	F	Silicone	Lips (unspecified)	8	NSAIDs, systemic and intralesional steroids	Resolved
Maly 38	2002	Israel	32	F	Silicone	Upper lip	24	–	Not reported
Ficarra 16	2009	Multiple	5656	F	Silicone	Both lips	120	NSAIDs, systemic steroids	Persistent
			34	M	Silicone	Upper lip	84	Surgical	Resolved
			50	F	Silicone	Lower lip	60	Intralesional steroids	Stable (asymptomatic and no change)
			39	F	Silicone	Lower lip	84	Surgical	Stable
			38	F	Silicone	Lower lip	60	Surgical	Resolved
			52	F	Silicone	Upper lip	12	Surgical	Lost to follow-up
			77	F	Silicone	Upper lip	180	Surgical	Lost to follow-up
Alijotas-Reig 39	2009	Spain	65	F	PLLA	Both lips, NLF	15	HCQ, systemic steroids, NSAIDs	Remission
			60	F	PLLA	Both lips, NLF	60	Intralesional steroids, HCQ, allopurinol, prednisone, minocycline	Minor bouts
			48	F	PLLA	Lips (unspecified)	10	Intralesional steroids	Remission
			39	F	PLLA	Lips (unspecified)	6	Intralesional steroids, NSAIDs	Recurrent bouts
			59	F	PLLA	Both lips, NLF	17	Intralesional steroids, NSAIDs	Remission
Akrish 40	2009	Israel	41	F	PAIG	Lips (unspecified)	12	Surgical	Not reported
			43	F	PAIG	Upper lip	−	Surgical	Not reported
Baumann 41	2003	United States	31	F	Silicone	Both lips	6	Imiquimod topical cream	Resolved
Da Costa Miguel 42	2009	Brazil	56	F	PMMA	Lips (unspecified)	12	Surgical	Not reported
Dionyssopoulos 7	2007	Multiple	45	F	PLLA	Both lips	4	Intralesional steroids	Significant volume reduction, granulomas not completely resorbed
Friedmann 43	2016	Multiple	46	F	Silicone	Both lips, NLF	12	Intralesional 5-FU	Significant reduction in lesion size/firmness
			47	F	Silicone	Upper lip	120	Intralesional 5-FU	Progressive improvement
Weyand 44	2008	Germany	62	F	Mixed (HA + HEMA + EMA)	Lips (unspecified)	0	Surgical, antibiotics, intralesional steroids	Persisted (physical + psychological complications)
Grippaudo 9	2014	Italy	28	F	Silicone	Lips (unspecified)	12	Antibiotics	Resolved
			58	F	Mixed (silicone + HA + EMA + HEMA)	Lips (unspecified)	–	Antibiotics	Resolved
			34	F	PAAG	Lips (unspecified)	0.25	Surgical	Resolved
			45	F	PAAG	Lips (unspecified)	24	Antibiotics, surgical	Resolved
			55	F	PAAG	Lips (unspecified)	0.25	Antibiotics, surgical	Resolved
			43	F	PAAG	Lips (unspecified)	72	Surgical	Resolved
			40	F	PAAG	Lips (unspecified)	36	Antibiotics, surgical	Resolved
			32	F	Mixed	Lips (unspecified)	12	Antibiotics	Resolved
			48	F	Mixed	Lips (unspecified)	60	Antibiotics, surgical	Resolved
			45	F	Collagen	Lips (unspecified)	12	Systemic steroids	Resolved
			38	F	PAIG	Lips (unspecified)	60	Systemic steroids	Resolved
			73	F	PAIG	Lips (unspecified)	36	Filler for asymmetry	Resolved
			28	F	PAIG	Lips (unspecified)	24	Surgical	Resolved
			55	F	PAIG	Lips (unspecified)	60	Antibiotics, surgical	Resolved
Sanchis-Bielsa 12	2009	Spain	63	F	Silicone	Lips (unspecified)	168	Systemic steroids	Partial healing
			70	F	Collagen	Lips (unspecified)	24	Systemic steroids	Resolved
			71	F	Silicone	Lips (unspecified)	2	Systemic steroids	Not resolved
			54	F	Silicone	Lips (unspecified)	1	Systemic steroids	Partial healing
Martin 45	2018	United Kingdom	24	F	HA (unspecified)	Upper lip	NA	NA	NA
			43	F	HA (unspecified)	Upper lip	NA	NA	NA
			67	F	HA + acrylic	Lower lip	NA	NA	NA
			62	F	HA + acrylic	Upper lip	NA	NA	NA
			44	F	Silicone	Lower lip	NA	NA	NA
			36	F	CaHA	Lower lip	NA	NA	NA
			48	F	Collagen	Lower lip	NA	NA	NA
			36	F	Silicone	Upper lip	NA	NA	NA

Abbreviations: CaHA, calcium hydroxyapatite; EMA, ethyl-methacrylate; F, female; FU, fluorouracil; HA, hyaluronic acid; HEMA, hydroxyl-ethyl-methacrylate; M, male; NA, not available or reported; PAAG, polyacrylamide gel; PAIG, polyalkylimide gel; PLLA, poly-L-lactic acid; PMMA, polymethylmethacrylate.

### Case Characteristics

There were 67 (98.5%) females and one (1.5%) male described in our cases. The average age was 50.0 years (range: 23–77 years). The mean time of onset of masses or nodules was 34.4 ( *n* = 55) months. Most patients presented with swelling, asymmetry, or erythema of their lips. Twenty-six cases (38.2%) presented with multiple nodules, 19 (27.9%) reported single nodules, and 23 cases (33.8%) did not report on the number of nodules. Masses were frequently described as discrete, indurated, mobile, firm, and slowly growing.

A histological analysis of 37 cases was reported. Thirty-one (83.8%) of these confirmed a foreign-body granuloma. The other cases reported extensive or chronic inflammation, sarcoid-like reaction, and a pseudocystic, fibrous-structure-containing translucent, viscous material that stained positive for Alcian blue (a marker for HA).

**Fig. 4:**
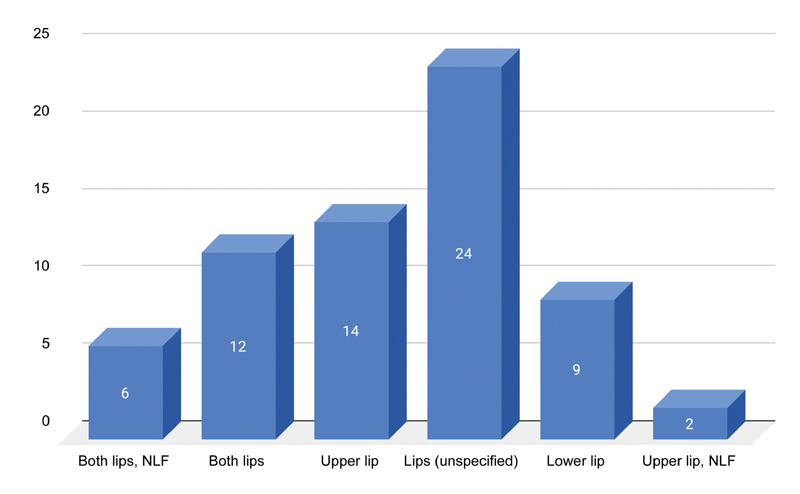
Initial site of injection for augmentation.

Only one case reported on the injection volume, which was 3 mL for both lips. 7 No studies reported on the method of injection or qualifications of the injector. Regarding the initial treatment area for augmentation, many studies reported injecting in the “lips” and did not specify if it was both lips and one lip. The frequency of other injected areas is displayed in Fig. 4 .

### Product Used

**Fig. 5:**
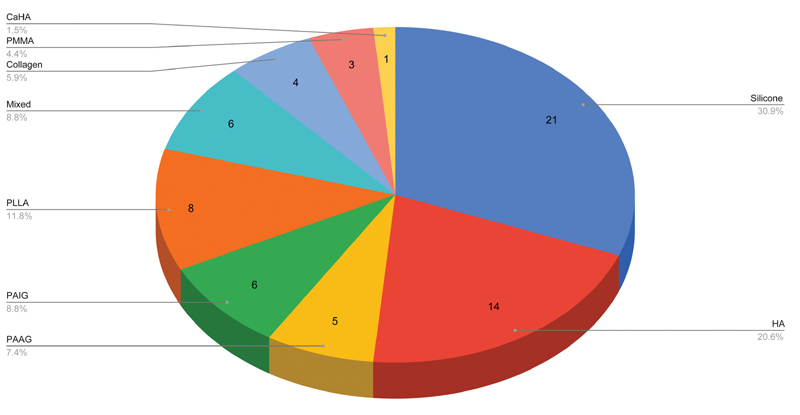
Reported filler type used. CAHA, calcium hydroxy apatite; HA, hyaluronic acid; Mixed, combination of HA and hydroxy-ethyl-methacrylate (HEMA) and ethyl-methacrylate (EMA); PAAG, polyacrylamide gel; PAIG, polyalkylimide gel; PLLA, poly-L-lactic acid, PMMA, polymethyl-methacrylate microspheres.

The most commonly reported dermal filler product used was silicone (21/66, 31.8%) followed by HA (14/66, 21.2%). Various other dermal fillers were used ( Fig. 5 ).

### Treatment

**Fig. 6:**
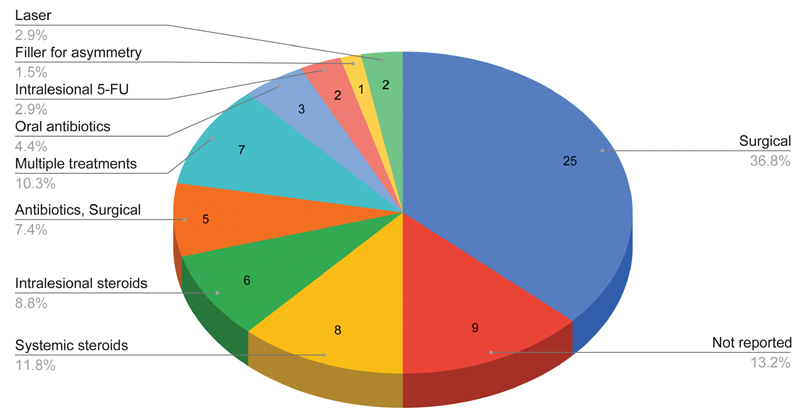
Treatment of cases. FU, fluorouracil.

Treatment typically consisted of oral antibiotics, intralesional steroids, or oral steroids ( Fig. 6 ). For instance, Alijotas-Reig et al initially treated a case of multiple nodules with antibiotics (quinolones) and nonsteroidal anti-inflammatory drugs (NSAIDs), which had no effect. 8 However, when oral prednisone and hydroxychloroquine (400 mg/day) were added, many of the nodules resolved. Grippaudo et al described a case with “multiple angry red lumps” 12 months following lip augmentation, which was successfully treated with three rounds of antibiotics. 9 Curi et al reported a non-well-defined nodule which was initially evaluated by a punch biopsy. This identified the foreign granuloma which was successfully treated with oral steroids for 2 months. 10 Goldman and Wollina reported granulomas after PMMA injection which was first treated by intralesional 1,064 nm Nd:YAG laser in combination with suction using a blunt liposuction cannula either alone or combined with surgery. 11 Sanchis-Bielsa et al described a nodule with associated swelling that partially resolved with oral steroids (30–90 mg/day) for 10 to 15 days. 12 Surgical treatment was often offered to those with persistent nodules (25/66, 37%), which led to complete resolution of the nodule.

### Outcomes

A summary of the outcomes is reported in Table 3 . In most cases, there was resolution of the nodule(s) or remission (42/68, 61.8%). Eight cases reported only partial healing or persistent disease. Two cases reported a significant reduction in lesion size. Seventeen cases were lost to follow-up or did not report on the outcome.

**Table 3:** Outcomes of reported cases

Outcome	Number of cases (%)
Resolved or remission	42 (61.8%)
Incomplete resolution (partial, persistent disease, minor bouts)	7 (10.3%)
Significant reduction in lesion size or progressive improvement	3 (4.4%)
Lost to follow-up	3 (4.4%)
Not reported	13 (19.1%)

## Discussion

Lip augmentation with dermal fillers is rising in popularity. This systematic review analyzed reported cases of granulomas or nodules secondary to any dermal filler for lip augmentation. Of the 66 cases, 31 (47.0%) confirmed the presence of a delayed-onset foreign-body granuloma. Of note, not all nodules are considered granulomas.

Nodules following treatment of filler are commonly categorized as inflammatory or noninflammatory in origin. 13 Inflammatory nodules may occur days to years after treatment as a result of host response to a foreign body. In contrast, noninflammatory nodules commonly occur immediately after treatment and are typically caused by improper placement of the filler material. These cases may not be reported in the literature as observation, massage, or hyaluronidase may resolve these nodules. Temporary and biodegradable HA should have a minimal foreign-body response compared with permanent and nonresorbable fillers such as silicone. 14 However, our study demonstrates that treatment with HA does not preclude a risk of granuloma formation. 15

Diagnosis of lip nodules can be challenging as patients may not associate them with filler treatment performed weeks, months, or years prior. A broad range of differential diagnoses commonly includes abscesses, sialadenitis, mucocele, benign salivary gland neoplasm, or malignancy. Infection can present early or late in the clinical course and are more commonly single nodules. The involvement of multiple sites more likely suggests a foreign-body granulomatous response. Timely and proper diagnosis of these masses is crucial as they may mimic a neoplasm, which is particularly important given the generally older age group of these patients.

Silicone liquid (dimethylpolysiloxane) has been widely used for soft tissue augmentation. When it was first introduced in the late 1950s, it was considered safe as it was not known to elicit pathological disease in humans. Additionally, non-medical-grade silicone fluid was used in many patients. Years later, the term “siliconoma” was coined to describe the granulomatous reactions in soft tissues of patients who had received liquid silicone injections. 16 The pathogenesis of silicone granuloma is unknown, but factors such as the volume of the injection, impurities present in the fillers, and the physical properties of fillers have been reported to affect granuloma formation. 17 Silicone granulomas have been reported in other areas of the face such as the eyelids and cheek and the onset ranged from 5 months to 15 years, which is similar to that of our observed cases. 18 19 Liquid silicone injections remain controversial, particularly in countries where there is inadequate control of quality of material used for soft tissue augmentation.

Several hypotheses have been suggested for the pathogenesis of granulomatous reactions to HA. HA is a polysaccharide that is fermented from bacteria and impurities from this process may elicit a hypersensitivity response, particularly in patients who have undergone repeated injections. 15 20 Additionally, during the production of HA filler products, stabilization through a cross-linking process occurs, which allows the product to be resistant to natural hyaluronidases. Over time, the breakdown and byproducts of the cross-linked material may induce a host inflammatory response. 21 Lastly, bacteria inoculated during the injection may form a biofilm. The biofilm surrounding the HA material creates a matrix that can inhibit natural hyaluronidases from degrading the HA. These biofilms can induce a minimal infection with little host response, making them asymptomatic for months or even years. 22 In our cases, microscopic examination of the nodules confirmed the presence of HA years following treatment, indicating failure in the degradation process. A subsequent delayed foreign-body tissue reaction to biofilm could have been elicited in the months or years following initial injection.

Restylane was the most common HA filler used in our reported cases. One explanation of this finding may be due to the rheological properties (i.e., cohesion) or processing technologies of the fillers. Popular Restylane products for lip augmentation are non-animal HA, while Juvéderm uses Hylacross and Vycross cross-linking technologies. 23 Bhojani-Lynch reported a case where two different brands (Teosyal Puresense Ultra Deep and Belotero Intense) were injected into various parts of the face during the same session and only areas treated with Teosyal triggered a hypersensitivity reaction characterized by diffuse redness and swelling without lumps. The authors suggest that reactions to HA fillers may be attributed to rheological or processing technologies of the fillers. 24 Additionally, more reports of Restylane may be published as it was Food and Drug Administration-approved and more widely used earlier than Juvéderm and Belotero. In our cases, granuloma formation was more likely found in the upper lip compared with the lower lip, which may be due to the fact that the upper lips are more commonly treated. While our review includes studies involving granuloma formation following the use of calcium hydroxyl apatite and PLLA, current standard practice does not use these products.

Many patients were initially treated with nonoperative methods such as NSAIDs, antibiotics, and systematic and/or intralesional steroids. Laser therapy has also been successfully used. 11 Goldman and Wollina reported the use of a subdermal, intralesional 1,064 nm neodymium-doped yttrium aluminum garnet (Nd:YAG) laser in combination with a blunt liposuction cannula suction in 81 patients with facial lumps or granulomas following PMMA. The procedure was well tolerated and 86.4% of the patients were satisfied. 25 In our subset of patients, many ultimately required surgical removal of the granuloma, particularly if there was a single nodule. For those with multiple nodules, more aggressive treatments were pursued, including intralesional 5-FU.

### Limitations

This systematic review has some limitations. The sample size is small with only 66 cases of dermal filler-related lip granuloma or nodules reported in the literature. There was also a lack of uniformity in describing the cases such as time of onset versus time the patient presented for care. There are many factors that determine the likelihood of dermal filler complications such as injector experience, training, and techniques used. However, this information was not available in any cases, potentially owing to the long duration from initial injection to time of onset of nodule(s). Some studies reporting on lip nodules or granulomas secondary in filler were excluded from our study because they did not provide enough patient case information. Therefore, our review may not have included all reported cases in the literature.

## Conclusion

Understanding the sequelae of lip augmentation with dermal filler products allows clinicians to provide safe and effective treatment. Nodules that present months to years following filler treatment may represent a foreign-body granuloma. A combination of oral antibiotics, intralesional or oral steroids, and surgical excision successfully treated the majority of cases in our study. Future studies evaluating the development of granulomas should include treatment injection methods and techniques to better elucidate potentially related causes.
